# Impact of change in coronal plane alignment of knee (CPAK) classification on outcomes of robotic-assisted TKA

**DOI:** 10.1186/s42836-024-00239-1

**Published:** 2024-04-04

**Authors:** Sarang Agarwal, Femi E. Ayeni, Rami Sorial

**Affiliations:** 1 Department of Orthopaedics, Nepean Hospital, Derby Street, Kingswood, NSW 2747 Australia; 2 Nepean Institute of Academic Surgery, Nepean Clinical School, The University of Sydney, 62 Derby Street, Kingswood, NSW 2747 Australia

**Keywords:** Robotic-assisted total knee arthroplasty, TKA, ROSA, Mechanical alignment, Coronal plane alignment of knee

## Abstract

**Background:**

Mechanical alignment with total knee arthroplasty (TKA) has been widely used since the implantation of the first prosthetic knee. Multiple studies have reported 80% patient satisfaction with TKA. However, the reported patients’ dissatisfaction is believed to be caused by having to convert different knee alignments to neutral alignments. It is postulated that a change in the CPAK classification of knees leads to patient dissatisfaction.

Thus, we hypothesized that a change in CPAK classification with robot-assisted TKA with mechanical alignment does not significantly lead to patient dissatisfaction.

**Methods:**

We retrospectively analyzed 134 patients who underwent robot-assisted mechanical alignment total knee arthroplasty (MA-TKA) using cementless implants and classified them into CPAK system pre- and post-operatively. One year after TKA surgery, we recorded binary responses to patients’ satisfaction with the outcome of surgery and analyzed if a change in CPAK classification is associated with the outcome of surgery.

**Results:**

We found that 125 out of 134 patients (93.28%) were happy with the outcome of surgery. CPAK classification was changed in 116 patients (86.57%) and maintained in 18 patients (13.43%). Our results also showed that 111 (95.7%) out of 116 patients who had a change in CPAK and 14 (77.8%) out of 18 patients who maintained their CPAK post-surgery were happy with the outcome of surgery (OR = 6.3, CI 1.741–25.17, *P* value = 0.019).

**Conclusion:**

We concluded that changing the patient’s native joint line and CPAK classification does not significantly change the outcome of surgery in terms of satisfaction. The dissatisfaction rate of 20% as published by previous researchers may be confounded by other reasons and not just due to changes in alignment and joint line.

## Introduction

Since the implantation of the first ivory hinge joint by Themistocles Gluck in the 1880s, there have been numerous changes in the total knee arthroplasty (TKA) implant design and the number of knee replacements has been increasing [1]. The total number of TKAs reported in the Australian joint registry is > 500,000 [2]. Even after improved surgical techniques and implant design, various studies have reported a 15%–20% patient dissatisfaction rate with TKA [3, 4].

Neutral mechanically aligned TKA has shown good survivorship and clinical outcomes for decades, however, a recent study has exhibited that only 5% of the population has a neutrally aligned native knee [5]. It is being hypothesized, that patient dissatisfaction arises due to forcing the knee into an unnatural alignment when performing a mechanical alignment total knee arthroplasty (MA-TKA) [6].

In pursuit of improving patient satisfaction rates, there has been a move towards restoring native, pre-arthritic alignment and joint obliquity of the knee [7]. Kinematic alignment (KA) method aims to replicate normal movement around three kinematic axes of the knee joint by restoring constitutional knee alignment and achieving balanced flexion–extension gaps with minimal periarticular soft-tissue release [8]. Macdessi et al. proposed a new classification to describe knee alignment in healthy and arthritic populations. Coronal Plane Alignment of Knee (CPAK) classification helps in determining the population that may benefit the most from kinematic alignment [7].

New techniques are being continuously developed for performing a successful TKA and to improve patient satisfaction. The next-generation robotic-arm assisted TKA (RA-TKA) was observed to have improved accuracy of bone cuts and alignments, as compared to the first-generation robot systems [9]. Several studies have shown that RA-TKA attains more accurate implant positioning and leads to lesser soft tissue trauma [10, 11]. However, long-term studies that determine clinical scores and happiness of patients with RA-TKA are not yet available.

Multiple studies that compared mechanical and kinematic alignment reported no significant difference in medium-term clinical scores, patient-reported outcome measures (PROMs) or patient dissatisfaction with TKA [12–14].

To the best of our knowledge, no other study has reported on the significance of the change in CPAK classification with subjective patient happiness with RA-TKA surgery and their outcomes. We conducted a retrospective study to analyze whether a change in CPAK classification is associated with a higher level of patient unhappiness following TKA surgery.

## Methodology

### Study design

This was a retrospective cohort analysis, reviewing the first 175 patients who underwent TKA using robotic technique with cementless femoral and tibial components by a single surgeon between December 2019 to July 2021. The primary outcome of this study was to correlate a binary response of patient being satisfied with the outcome of surgery to a change in their CPAK classification following RA-TKA. Secondary outcomes were any kind of intra- or postoperative complications and revision rates.

All patients without preoperative long leg X-rays or postoperative CT scans for Perth protocol, and those lost to follow-up were excluded from the study. Preoperative long leg X-rays for 6 patients couldn’t be accessed, 27 patients didn’t have postoperative CT scans and 8 patients were lost to follow-up. After all the exclusions, 134 patients were selected for the study. Preoperative and postoperative radiographic measurements of arithmetic hip-knee-ankle angle (aHKA) and joint line obliquity (JLO) were done and the correlation of CPAK with surgical outcomes was assessed.

### Radiographic measurements

All patients underwent long leg X-rays preoperatively as part of the imaging protocol for ROSA TKA (Zimmer Biomet, Warsaw, IN, USA), which included the hip, knee and ankle of the operative side. A single experienced observer not involved in the surgical procedures measured the lateral distal femoral angle (LDFA) and the medial proximal tibial angle (MPTA) on these radiographs. LDFA was measured as the lateral angle subtended by a line from the centre of femoral head to the centre of intercondylar notch and the other line tangential to the femoral condyles. MPTA was measured as the medial angle subtended by a line from centre of tibial spines to the centre of ankle joint and the other line tangential to the tibial articular surface. Preoperative aHKA was calculated by the formula MPTA-LDFA and JLO was the sum of MPTA and LDFA. Each knee was classified according to the CPAK system preoperatively. Postoperatively, scout images from CT Perth protocol were used to measure MPTA and LDFA and to classify patients into CPAK categories accordingly.

### Technique

All patients underwent MA-TKA using robot assistance performed by a single surgeon using cementless Zimmer Persona implants. Robot assistance was used in all surgeries, aiming to restore mechanical alignment for an HKA of 0 with a flexion gap balancing technique using the FuZion device (Zimmer Biomet, Warsaw, IN, USA). Surgeries were done using the standard medial parapatellar approach without a tourniquet.

The standard surgical exposure was just sufficient to expose the anterior aspect of the tibia to ensure juxtaposition of the tibial cutting block of the robot arm. Any further exposure of the anteromedial tibia subsequently was assessed as a medial release. All large and accessible osteophytes were removed. Femoral and tibial bone cuts were taken perpendicular to the mechanical axis (0 degrees varus-valgus). We aimed to ensure at least 19 mm of joint space in the medial compartment for a valgus deformity or the lateral compartment for a varus deformity. The opposite compartment was accepted at whatever the tighter joint space was observed, as the surgeon would then perform the required soft tissue releases to ensure balance after bone resections and removal of remaining osteophytes. Once the proximal tibial and distal femoral cuts were made and validated, the remaining osteophytes were removed, and the extension space was assessed with the spacer block. The aim was to ensure sufficient space in one compartment to accommodate the smallest spacer block and ensure extension was between 0 and 10 degrees. If the medial compartment was tight, then a posteromedial release would be completed as needed to effect balance and fully accommodate the spacer. If the lateral compartment was tight, then a fenestrated posterolateral capsular release plus or minus posterior cruciate ligament (PCL) sacrifice was completed as needed to effect balance and fully accommodate the spacer. These releases were documented. The FuZion device would then be used and tensioned in 95 degrees of flexion to balance the flexion space. This rotation would then be incorporated into the flexion balance algorithm, and final flexion balance confirmed ensuring at least 19 mm medial and lateral joint spaces in flexion before the final femoral bone cuts were completed, dictating the rotation of the femoral component independent of the transepicondylar or posterior condylar axes. The trial reduction was the final opportunity to ensure balance was achieved and if further releases were required. This was particularly relevant for sagittal balance and flexion range and, if required, the PCL might be fenestrated if any tibial lift-off was observed. After implantation of the true prosthesis, the final balance through the range was assessed and documented.

The surgical technique, method of closure and postoperative rehabilitation protocol were similar in all cases. Patients were followed up at 6 weeks, 6 months and then at the 1 year mark. All patients underwent knee CT scans according to Perth Protocol at 6–12 weeks post-surgery. Using the data from these scans, we calculated the final LDFA and MPTA. These values were then used to calculate postoperative aHKA and JLO. Patients were then reclassified according to the CPAK system. At the 1-year follow-up, an independent researcher asked the patients a question and a binary response (Yes or No) was noted.

“Are you happy with the outcome of your left/right knee replacement?”

Binary response of patient in terms of satisfaction with the outcome of surgery was recorded. Analysis of binary response was done with the CPAK classification before and after TKA and a literature review was done.

### Statistical analysis

We used descriptive statistics to summarize outcomes. The Chi-squared test was used for categorical variables and multivariate analyses to test which of the independent variables had a significant association with the patients’ assessment of TKA outcomes and happiness with TKA surgery. For all analyses, *P*-values of less than 0.05 were considered statistically significant. All analyses were performed by using GraphPad Prism Version 9.4.1 (681).

## Results

### Demographics and radiographic analysis

Our study population consisted of 101 females and 33 males. The median age of the patients was 70 years (age range 48–89 years) and 58 patients received left-sided TKA and 76 patients underwent right-sided TKA surgery. The most common CPAK type preoperatively was type 2 (27.61%). This was followed by type 1 (20.89%), type 5 (19.4%), type 3 (17.91%).

**Fig. 1:**
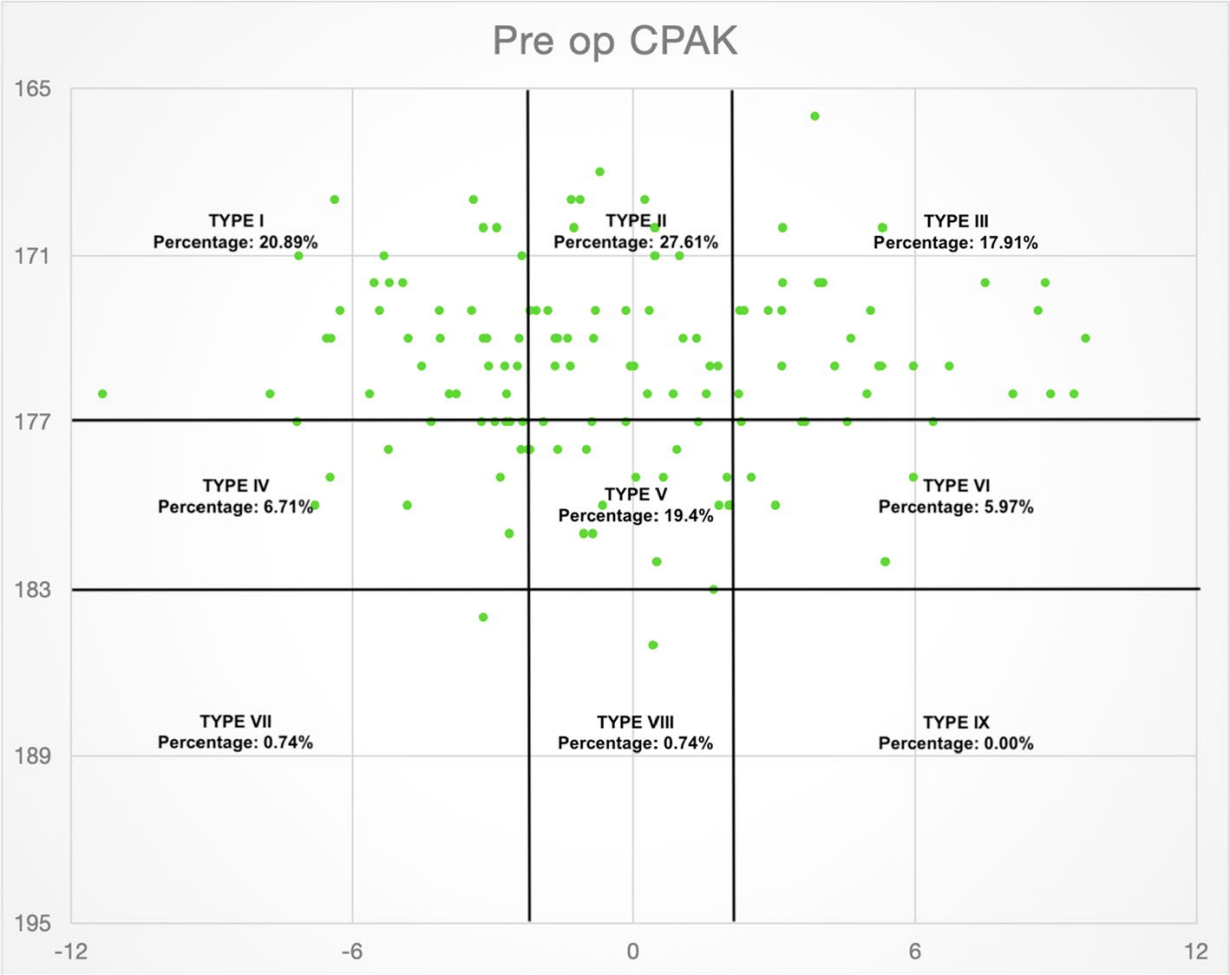
Scatter plot showing patients in various CPAK categories preoperatively

**Fig. 2:**
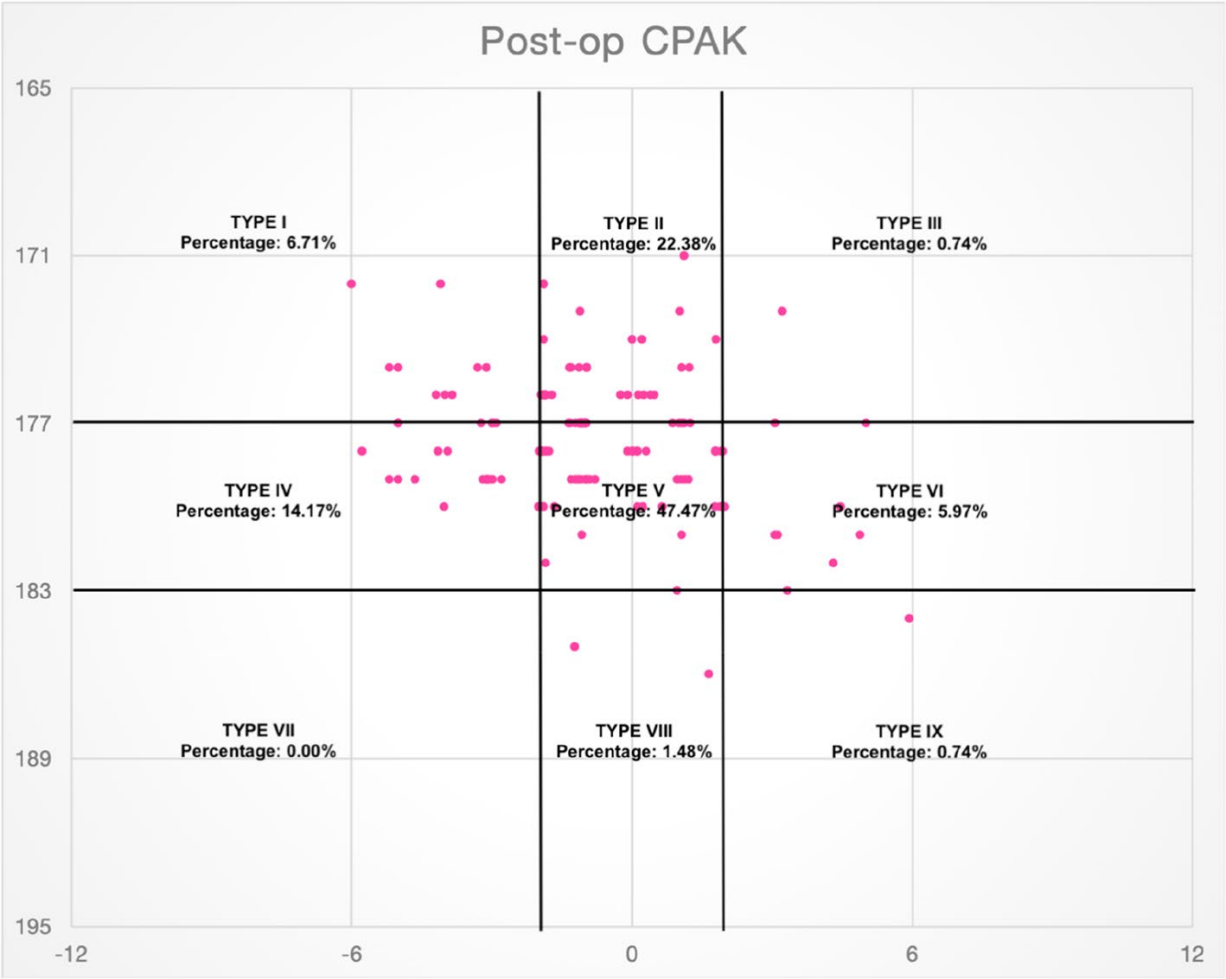
Scatter plot showing patients in various CPAK categories postoperatively

Only 2 patients were in the JLO apex proximal category (types 7, 8 and 9) with 0 patients in the CPAK type 9 category (Fig. 1). Postoperatively, CPAK type 5 was the most common group with 64 (47.47%) out of 134 patients. This was followed by CPAK type 2 (22.38%) and type 4 (14.17%) (Fig. 2).

### Outcomes and questionnaire responses

116 (86.56%) out of 134 patients had a change in CPAK classification after their TKA and 18 (13.44%) patients retained their preoperative CPAK type. As per the questionnaire, of the whole group, 125 out of 134 patients (93.28%) were happy with the outcome of surgery. Out of the 116 patients who had a change in CPAK with MA-TKA, 111 (95.69%) were happy with the outcome of surgery (*P* value = 0.019) and 14 (77.78%) out of 18 patients who maintained their CPAK post-surgery were happy with the outcome of surgery (*P* value = 0.019).

The mean preoperative flexion of patients was 101.56 degrees which increased postoperatively to an average of 117.91 degrees.

**Fig. 3:**
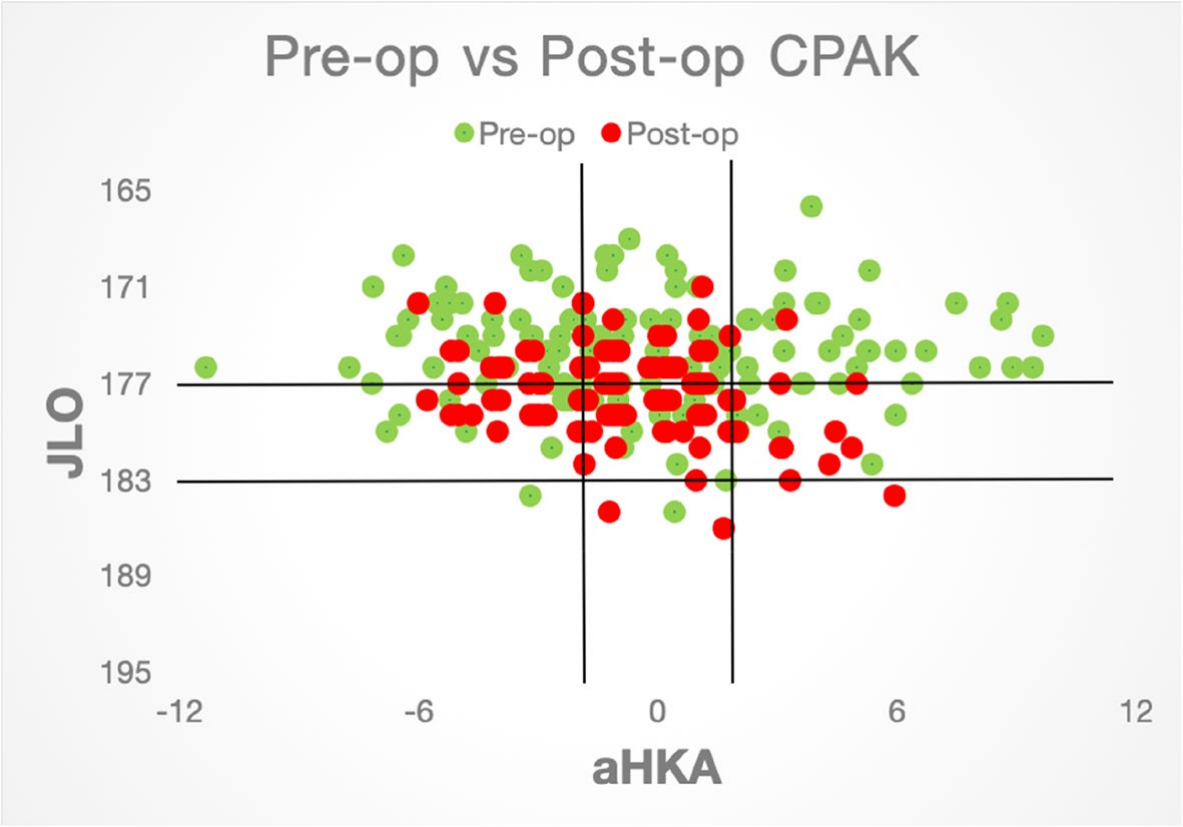
Scatter plot comparing the distribution of patients in various CPAK categories preoperatively and concentration in category 5 postoperatively as we aimed for mechanical alignment

However, no significant association was found between sex of the patient, preoperative flexion range, postoperative flexion range and side of limb operated to any of the primary outcomes on multivariate analyses (*P* value > 0.05), Table 1, (Fig. 3).

**Table 1:** Multivariate analysis of patient happiness with outcomes of TKA surgery

Variable	Odds Ratio	95% CI	*P*-value
Sex [F]	0.68	0.08775–3.331	0.6649
Side of limb operated [R]	1.34	0.3006–6.119	0.6940
Pre-op Flexion range	0.96	0.8799–1.031	0.2753
Post-op Flexion range	1.04	0.9366–1.136	0.4636
Change in CPAK [Y]	6.52	1.362–31.09	0.0158

### Complications and revision rates

There were no complications observed in our study population. We also conducted a review of our patients with an ad hoc report from the Australian Orthopaedic Association National Joint Replacement Registry (AOANJRR), and it was found that no patient from this study had undergone a revision procedure.

## Discussion

The development of CPAK classification by Macdessi et al. [7] in 2021 has contributed to an unending debate regarding the search of the perfect knee alignment. This classification divides the knee into 9 different phenotypes based on JLO and aHKA. The most common CPAK types, in order, were type 2, type 1, and type 5, with rarity in types 7, 8, and 9. Similar findings were seen in our study group as we classified the patients preoperatively into CPAK types. Hsu et al. [15] modified the existing CPAK classification by changing boundaries of aHKA and adding another variable aJLO to prevent concentration of Asian knee in a few phenotypes.

In 2019, Hirschmann et al. [16] studied coronal plane alignment of normal subjects and defined different global, femoral and tibial phenotypes. Jenny et al. [17] compared CPAK classification with the functional knee phenotype classification and found significant differences and a weak correlation between HKA and aHKA in the same individuals.

This shows non-uniformity and inconsistency in the validation of CPAK in different types of populations. Another shortcoming of CPAK classification is that it doesn’t take into account bony erosions and extra-articular bone deformities, which may lead to gross inaccuracy in measuring aHKA. This was overcome by Mullaji et al. who described different classifications for varus [18] and valgus knees [19], thus again showing non-uniformity of CPAK classification.

Since the advent of total knee arthroplasty, the concept of mechanical alignment of knee has been popular and well-reproduced with excellent results in terms of PROMS. The traditional standard of care in TKA is to restore the overall limb alignment to a neutral mechanical axis, as it has been postulated that mechanical alignment leads to the longest implant survival [20]. Kim et al. [21] showed that achieving better accuracy in terms of alignment and component positioning leads to improved survivability of implants.

This better accuracy has been achieved using robot-assisted TKA. Song et al. [22] concluded that RA-TKA leads to significantly less number of outliers in terms of postoperative limb alignment when compared with conventional instruments. Selvanathan et al. [23], in a prospective study with 175 patients, concluded that robot assistance enhances bone cut precision in TKA, which allows titration of soft tissue balance to achieve optimal balance. Interestingly, multiple studies have reported only 80% satisfaction rate with TKA [3, 4].

This has led to the popularity of KA-TKA and a number of other alternative alignment options being pursued. The principle of KA-TKA requires a component alignment to be done according to the natural knee alignment, thus replicating the preoperative CPAK phenotype. Placing all knees in mechanical alignment may lead to patient dissatisfaction. Recent studies [13, 24–26] have demonstrated no significant difference in pain characteristics, PROMS and functional outcomes in KA-TKA with respect to MA-TKA. These findings are similar to our study in which there was no association between patient satisfaction and change in the type of CPAK.

In our study, we reported that 93.28% of patients were happy with the outcome of their TKA surgery, irrespective of the change in CPAK types and postoperative flexion range. These findings were similar to what has been reported by Klem et al. [27], who identified three pathways through which patients reached different levels of high and low satisfaction. The author concluded that preoperative education could moderate unrealistic patient expectations and high hopes which can result in increased postoperative patient satisfaction with surgery. Mancuso et al. [28] reported a case–control study using a similar approach, whereby preoperative educational classes moderated preoperative expectations of recovery after TKA or THA, thus translating into better postoperative hip and knee scores. Interestingly, there were no complications reported in our study population, 9 patients who were dissatisfied with the outcome of TKA did not show any concerning features on follow-up, such as loosening, gross instability, infection, fracture. We did not include any objective scoring system in our study.

The present study has a few limitations. First and the most important one was the non-utilization of standardized scores, such as Knee Injury and Osteoarthritis Outcome Score (KOOS), Oxford Knee Score (OKS), International Knee Documentation Committee (IKDC), Knee Society Score (KSS) for determining our primary outcomes. These scores have drawbacks despite being standardized in the sense that patient’s happiness is subjective and using the scores can be arbitrary to assess happiness in this situation. Bullens et al. [29] showed that there was no correlation between objective and subjective patient outcome scores, indicating that surgeons and patients have different criteria for satisfactory outcomes after TKA. Another limitation of our study was that we used binary response to a question, which is not a standardized method of outcome assessment but subjective scales like Likert satisfaction scale have their demerits. Likert satisfaction scale has five different responses without distinct definitions which can create confusion amongst patients and it also requires a certain level of vocabulary to understand each response. This can confound the results of the study.

We believe, that individual expectations with surgery are different and a binary response in terms of happiness can more accurately describe patients’ satisfaction, the investigator did not ask about the reason for dissatisfaction during the phone interviews. Our follow-up period and response to the questionnaire was 1 year. A longer follow-up period with a delayed survey would be more robust to reap long-term results, however, we noted that patient happiness at 1 year may be extrapolated to a longer term.

## Conclusion

Kinematic or functional alignment in TKA is based on philosophies of minimal soft tissue releases and replicating the original phenotype of the knee. But we can safely conclude, through this study, that changing CPAK classification from one phenotype to another does not result in patient unhappiness with the outcome. The change in CPAK classification post-TKA is not the reason that the referred 20% dissatisfaction may occur following MA-TKA. Rather, a well-balanced knee, done by either kinematic or mechanical alignment philosophies should be the target of modern TKA.

## Data Availability

The data that support the findings of this study are available from Nepean Blue Mountain Local Health District, but restrictions apply to the availability of these data, which were/are used under license for the current study, and so are not publicly available. Data are however available from the authors upon reasonable request and with permission of Nepean Blue Mountain Local Health District.
